# Serum cholesterol and testicular cancer incidence in 45 000 men followed for 25 years

**DOI:** 10.1038/sj.bjc.6602539

**Published:** 2005-04-12

**Authors:** A-B Wiréhn, S Törnberg, J Carstensen

**Affiliations:** 1 Department of Health and Society, Linköping University, SE-581 83 Linköping, Sweden; 2 Cancer Screening Unit, Oncologic Centre, Karolinska University Hospital, SE-171 76 Stockholm, Sweden

**Keywords:** epidemiology, testicular neoplasm, cholesterol

## Abstract

In a 25-year follow-up study of 44 864 men with measured serum cholesterol levels, the testicular cancer hazard ratios for the serum cholesterol categories 5.7–6.9 and ⩾7.0 mmol l^−1^ *vs* the reference category (<5.7 mmol l^−1^) were 1.3 and 4.5, respectively; *P*-value for trend=0.005. This highly significant association suggests that high-serum cholesterol is a risk factor for testicular cancer.

An increased cancer risk has been reported at both high (e.g. Törnberg *et al*, 1986; Yamada *et al*, 1998) and low (e.g. Eichholzer *et al*, 2000) serum cholesterol concentrations. To our knowledge, the relation between testicular cancer risk and serum cholesterol has not been evaluated. Testicular cancer is the commonest cancer site in men aged 15–44 years in Sweden as well as in many other countries. The incidence of testicular cancer has increased in recent decades although the incidence is relatively low, 5.8 per 100 000 in Sweden 2002. Nondescended testes at birth and other abnormal testicular developments are well-established risk factors for testicular cancer (Scottenfeld and Fraumeni, 1996, pp 1213). An ecological correlation between testicular cancer and fat consumption has been demonstrated (Armstrong and Doll, 1975) and case–control studies have also shown an increased risk associated with a high intake of total fat, saturated fat, dietary cholesterol (Sigurdson *et al*, 1999) and dairy products (Davies *et al*, 1996; Garner *et al*, 2003). We have investigated the relation between serum cholesterol and testicular cancer using data from the Värmland cohort.

## MATERIALS AND METHODS

Between 1963 and 1965 a mass screening health trial, ‘the Värmland survey’, was conducted in Sweden for a large cohort of 92 710 individuals aged 17–74 years to identify early-stage diseases in an unselected population (Törnberg *et al*, 1989). Among other measures, blood chemistry analysis including non-fasting serum cholesterol was included in the survey. Earlier findings based on the data from this cohort (Törnberg *et al*, 1989) accorded with results of other studies, such as the well-known association between serum cholesterol level and coronary heart disease.

The national registration system in Sweden using personal code numbers for all residents makes it possible to link registries to one another. Thus, the cohort data were matched with the Swedish Cancer Registry and the Swedish Cause of Death Registry. Matched data were examined for records of cancer registration and death between 1958 and 1987, giving a 25-year period of follow-up. A more detailed delineation of the cohort has been reported elsewhere (Törnberg *et al*, 1989).

Subjects with reported cancer (at any site) before they were examined within the survey were excluded from the study population. To avoid the possibility of incorrect conclusions because of inverse causality, that is, that the result is a reflection of preclinical testicular cancer, the cases that occurred within 2 years from the start of the follow-up period were excluded. For statistical analysis, Cox's proportional hazard model was used, with months from serum cholesterol test to testicular cancer event recorded as the follow-up time variable. Observations with no cancer event were censured at the time of death or end of the follow-up period. Hazard ratio (HR) estimates with 95% confidence intervals (CI) and two-tailed statistical tests of significance were computed using the Cox's regression model. In the analysis, serum cholesterol was classified into three categories, namely, serum cholesterol level <5.7, 5.7–6.9 and ⩾7.0 mmol l^−1^. In the fitted regression model, the cholesterol categories were treated as an indicator variable, with the lowest category as a reference group. The regression model was adjusted by age (5-year groups). A trend test was also included in the analysis.

## RESULTS

Among the 44 864 men at risk in the cohort, there were 21 cases of testicular cancer during the follow-up period. A positive correlation between serum cholesterol level and testicular cancer incidence was found and the estimated HRs for the middle and highest serum cholesterol categories compared to the lowest was 1.3 (95% CI: 0.3–5.1) and 4.5 (95% CI: 1.3–16.2), respectively (*P*=0.005) (Table 1).

## DISCUSSION

Since high intake of saturated fat or meat is known to elevate the serum cholesterol concentration (Thorogood *et al*, 1990), the results corroborate the hypotheses advanced concerning a relation between fat intake and testicular cancer (Armstrong and Doll, 1975; Davies *et al*, 1996; Sigurdson *et al*, 1999; Garner *et al*, 2003). Nevertheless, the interpretation of the cholesterol–testicular cancer association may be complicated by other influencing and confounding factors. One example is under-nutrition, the essence of the ‘foetal origins’ hypothesis, which suggests that several adult diseases may be caused by under-nutrition *in utero* (Godfrey and Barker, 2000). In conformity with this hypothesis, studies have shown that high cholesterol levels (Davies *et al*, 2004) and testicular cancer (Akre *et al*, 1996; Moller and Skakkebaek, 1997) are associated with low birth weight. Available data suggest that serum cholesterol concentration in Swedish men decreased during the final decades of the past century (Jansson *et al*, 2003). Thus, changes in serum cholesterol concentration do not explain the increasing incidence rate of testicular cancer in recent decades.

In conclusion, the highly significant positive association between serum cholesterol and testicular cancer risk found in this population-based cohort study suggests that an elevated concentration of serum cholesterol is a risk factor for testicular cancer. However, since the finding is the first of its sort and because of the wide CIs, more data from other cohorts are needed to confirm the association.

## Figures and Tables

**Table 1:** Testicular cancer incidence in relation to serum cholesterol in a 25-year follow-up study of the Värmland cohort (*n*=44 864)

**Serum cholesterol categories (mmol l^−1^)**	**No. of cases**	**Hazard ratio**	**95% Confidence interval**
<5.7^a^	3	1.0	—
5.7–6.9	7	1.3	0.3–5.1
⩾7.0	11	4.5	1.3–16.2

a Reference category.

*P*-value for the trend=0.005.
